# A Mixture Model for Robust Point Matching under Multi-Layer Motion

**DOI:** 10.1371/journal.pone.0092282

**Published:** 2014-03-21

**Authors:** Jiayi Ma, Jun Chen, Delie Ming, Jinwen Tian

**Affiliations:** 1 National Key Laboratory of Science & Technology on Multi-Spectral Information Processing, School of Automation, Huazhong University of Science and Technology, Wuhan, Hubei, China; 2 Department of Electronics and Information Engineering, Huazhong University of Science and Technology, Wuhan, Hubei, China; University of Manchester, United Kingdom

## Abstract

This paper proposes an efficient mixture model for establishing robust point correspondences between two sets of points under multi-layer motion. Our algorithm starts by creating a set of putative correspondences which can contain a number of false correspondences, or outliers, in addition to the true correspondences (inliers). Next we solve for correspondence by interpolating a set of spatial transformations on the putative correspondence set based on a mixture model, which involves estimating a consensus of inlier points whose matching follows a non-parametric geometrical constraint. We formulate this as a maximum a posteriori (MAP) estimation of a Bayesian model with hidden/latent variables indicating whether matches in the putative set are outliers or inliers. We impose non-parametric geometrical constraints on the correspondence, as a prior distribution, in a reproducing kernel Hilbert space (RKHS). MAP estimation is performed by the EM algorithm which by also estimating the variance of the prior model (initialized to a large value) is able to obtain good estimates very quickly (e.g., avoiding many of the local minima inherent in this formulation). We further provide a fast implementation based on sparse approximation which can achieve a significant speed-up without much performance degradation. We illustrate the proposed method on 2D and 3D real images for sparse feature correspondence, as well as a public available dataset for shape matching. The quantitative results demonstrate that our method is robust to non-rigid deformation and multi-layer/large discontinuous motion.

## Introduction

Establishing reliable correspondence between two images is a fundamental problem in computer vision and it is a critical prerequisite in a wide range of applications including structure-from-motion, camera self-calibration, tracking, image retrieval, and object recognition [1]. In this paper, we formulate it as a matching problem between two sets of discrete points where each point is an image feature, extracted by a feature detector, and has a local image descriptor (e.g., SIFT [2]). The matching problem is ill-posed and is typically regularized by imposing two types of constraints: (i) a descriptor *similarity constraint*, which requires that points can only match points with similar descriptors, and (ii) *geometric constraint*, which requires that the matches satisfy an underlying geometrical requirement, which can be either parametric (e.g., rigid transformations) or non-parametric (e.g., non-rigid). Even after regularization there remain an exponential number of possible matches between the two sets and efficient algorithms are required to obtain the best solution by removing the false matches. The difficulty of the matching problem is typically made harder by the presence of unmatched points in the two images (due to occlusion or failures of the feature detectors).

A popular strategy for solving the matching problem is to use a two stage process. In the first stage, a set of *putative correspondences* are computed by using a similarity constraint to reduce the set of possible matches. This putative correspondence set typically includes most of the true matches, the *inliers*, but also a large number of false matches, or *outliers*, due to ambiguities in the similarity constraints (particularly if the images contain repetitive patterns). The second stage is designed to remove the outliers and estimate the inliers and the geometric parameters [3]–[5]. This strategy is commonly used for situations where the geometrical constraints are parametric, such as requiring that corresponding points lie on epipolar lines [1]. Examples of this strategy include the RANSAC algorithm [3] and analogous robust hypothesize-and-verify methods [4], [6], [7]. Although these methods are very successful in many situations they have had limited success if the geometrical constraints are non-parametric, for example if the real correspondence is non-rigid, and they also tend to degrade badly if the proportion of outliers in the putative correspondence set becomes large [5].

Recently, some new non-parametric model-based methods have also been developed to deal with the non-rigidity, such as identifying point correspondences by correspondence function (ICF) [5], vector field consensus (VFC) [8], [9], mismatch removal via coherent spatial mapping [10], as well as 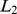-Minimizing Estimate-based method (RPM-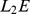) [11]. These methods works well when the scene contains some deformable objects. However, since they fit a smooth transformation for the scene motion, when the motion contains large discontinuities or multi-layer, for example, large depth discontinuities or motion inconsistencies, the smoothness prior will be violated and hence these methods will be badly degraded. Some graph matching based methods such as dual decomposition method [12] and graph shift (GS) [13] have also been proposed to capture different layers of motions, and hence robust to the motion with large discontinuities. However, these methods could not handle the 3D case.

**Figure 1:**
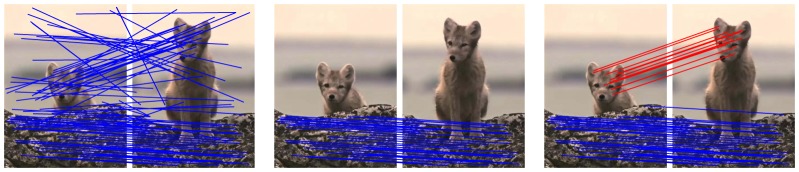
Schematic illustration of our approach for point matching. Left: a set of putative correspondences computed by SIFT matching. Middle: point matching results of a non-parametric model-based method, e.g., VFC [8]. Right: point matching results of our approach; different color indicates different component of the mixture model in our approach.

In this paper, we generalize the former non-parametric model-based methods to deal with large discontinuities. Rather than these methods which interpolates a global transformation, our approach uses a mixture model and fits the correspondences with a set of transformations. This enables us to capture multi-layer motion, and hence robust to large discontinuous motion. To illustrate the main idea of our approach, we show a simple example in Fig. 1. Given two sets of interest points extracted from an image pair, we want to match them to establish their point-wise correspondence. We first compute a set of putative correspondences based on their SIFT features as shown in the left of Fig. 1, which contains a number of outliers. Due to the motions of the fox and the ground are quite different in the scene, it is hard to fit a global transformation smooth enough on all the inlier correspondences. Therefore, the non-parametric model-based methods such as VFC [8] will only preserve the majority of the inliers which locate on the ground, as shown in the middle of Fig. 1. However, our approach fits multiple transformations, which can capture multi-layer motion, and hence both the inliers on the fox and the ground will be preserved, as shown in the right of Fig. 1.

## Related Work

This section briefly reviews the background material that our work is based on. This includes methods for establishing a set of putative correspondences and methods like RANSAC which use robust criteria for performing correspondence assuming parametric geometric constraints. Next we discuss approaches for solving matching problems which solve for a correspondence matrix between point sets.

There has been considerable study of robust estimation in the statistics literature [14], [15]. This work shows, for example, that maximum likelihood estimator of parameters using quadratic 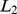 norms are not-robust and highly sensitive to outliers. By contrast, methods which minimize 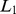 norm are more robust and capable of resisting a larger proportion of outliers. A particularly robust method is the redescending M-estimator [14]. It can be shown that this estimator results from using an explicit variable to indicate whether data is an outlier or an inlier (this indicator variable must be estimated).

The RANSAC algorithm matches two point sets by first computing a putative set and then using robust methods to impose parametric geometric constraints [3]. RANSAC uses a hypothesize-and-verify framework. It proceeds by repeatedly generating solutions estimated from a small set of correspondences randomly selected from the data, and then tests each solution for support from the complete set of putative correspondences. RANSAC has several variants such as MLESAC [4], LO-RANSAC [16] and PROSAC [6]. MLESAC adopts a new cost function using a weighted voting strategy based on M-estimation and chooses the solution that maximizes the likelihood rather than the inlier count. RANSAC is also enhanced in LO-RANSAC with a local optimization step based on how well the measurements satisfy the current best hypothesis. Alternatively, prior beliefs are assumed in PROSAC about the probability of a point being an inlier to modify the random sampling step of the RANSAC. A detailed comparative analysis of RANSAC techniques can be found in [7].

In the recent past, some new non-parametric model-based methods have also been developed, such as ICF [5], VFC [8], [17], RPM-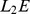 [11]. The ICF rejects outliers by learning a correspondence function pair which maps points in one image to their corresponding points in another. While the VFC converts the outlier rejection problem into a robust vector field interpolation problem which interpolates a non-parametric smooth motion field to fit the potential inliers. Similar to VFC, the RPM-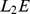 also fits a non-parametric spatial transformation, and the difference is that it uses a robust estimator to deal with outliers rather than explicitly modeling the outlier distribution in VFC.

Another strategy for point correspondences is to formulate this problem in terms of a correspondence matrix between points (in the two datasets) together with a parametric, or non-parametric, geometric constraint [18]–[22]. These approaches relate closely to earlier work on mathematical models of human perception of long-range motion. This includes Ullman's minimal mapping theory [23] and Yuille and Grzywacz's motion coherence theory [24] which formulate correspondence in terms of vector field interpolation and use Gaussian kernels. The iterated closest point (ICP) algorithm [18] is one of the best known point correspondence/registration approaches. It uses nearest-neighbor relationships to assign a binary correspondence, and then uses estimated correspondence to refine the transformation. Efficient versions of ICP use sampling processes, either deterministic or based on heuristics [25]. The nearest point strategy of ICP can be replaced by soft assignments within a continuous optimization framework, e.g., the TPS-RPM [20], [26]. In the recent past, the point registration is typically solved by probabilistic methods [21], [22], [27], [28]. The kernel correlation based method [27] models each one of the two point sets by two probability distributions and measures the dissimilarity between the two distributions. It was later improved in [22]. In [21] as well as in [22] and [28], the Gaussian mixture model is used to recast the point-to-point assignment problem into that of estimating the parameters of a mixture. This is done within the framework of maximum likelihood and the expectation-maximization (EM) algorithm [29].

Point correspondence has also been formulated as a graph matching problem, such as the dual decomposition (DD) [12], Spectral Matching (SM) [30], and graph shift (GS) [13], [31]. The DD approach formulates the matching task as an energy minimization problem by defining a complex objective function of the appearance and the spatial arrangement of the features, and then minimizes this function based on the dual decomposition approach. The SM method uses an efficient spectral method for finding consistent correspondences between two sets of features. Based on the SM method, the GS method constructs an affinity graph for the correspondences, and the maximal clique of the graph is viewed as spatially coherent correspondences. The SIFT-flow algorithm [32] builds a dense correspondence map between two arbitrary images with a particular advantage for matching two scenes; it does not explicitly deal with the outliers and may not be able to produce the accuracy for the precise matching for problems like structure-from-motion. Note that this type of graph matching formulation can in some cases be mathematically equivalent to the methods with correspondence variables and geometric constraints [24].

## Method

### Problem Formulation

Suppose now we are given a set of putative image point correspondences 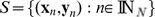, which may be perturbed by noise and outliers. The non-parametric model-based methods [8], [11] aim to fit a non-parametric transformation 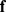 to the underlying inliers, i.e., 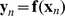 for any inlier 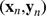, and hence remove outliers. The transformation 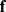 here is continuous and smooth, which is not able to handle multi-layer motion. In this case, a more robust method is desirable to provide stable results. To this end, we consider a mixture model and fit the correspondences with a set of transformations 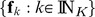 instead of just one global transformation.

We make the assumption that, for the inliers, the noise is Gaussian on each component with zero mean and uniform standard deviation 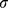; for the outliers, the output space is a bounded region of 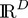 (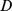 is the dimension of the data, typically 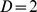 or 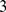), and the distribution is assumed to be uniform 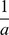 with 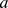 being a constant. Let us introduce a set of latent variables 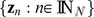, where 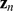 has an 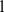-of-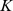
+1 representation in which a particular element 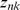 is equal to 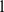 and all other elements are equal to 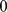. More specifically, the notation 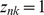 (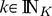) means that the 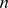-th correspondence is an inlier and it corresponds to the transformation 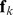, while 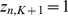 means that the 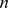-th correspondence is an outlier. The marginal distribution over 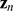 is specified in terms of the mixing coefficients 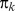, such that
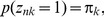
(1)where the parameters 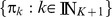 satisfy 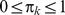 together with
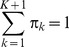
(2)in order to be valid probabilities. The likelihood of a correspondence 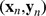 corresponding to the transformation 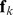 is drawn from a Gaussian distribution with mean 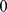 and covariance 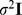:
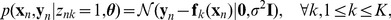
(3)where 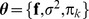 includes a set of unknown parameters. We denote the set of all observed data by 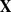 and 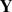, in which the 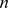-th rows represent 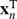 and 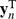 respectively, and similarly we denote the set of all latent variables by 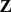. By making the i.i.d. data assumption, the likelihood is a mixture model given by
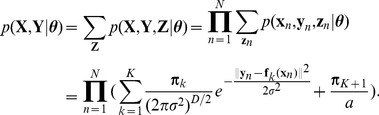
(4)distribution function is nonzero only in a bounded region (here we omit the indicator function for clarity).

We want to recover the transformations 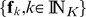 from the data 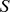. Taking a probabilistic approach, we assume 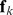 to be a realization of a random field with a known prior probability distribution 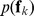. The prior is used to impose constraints on 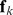, assigning significant probability only to those functions that satisfy those constraints. We consider the slow-and-smooth model [33] which has been shown to account for a range of motion phenomena, the prior of 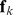 then has the form:
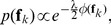
(5)where 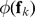 is a smoothness functional and 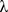 a positive real number (we will discuss the details of 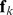 later). By applying Bayes rule, we seek a MAP solution of 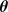, i.e.,
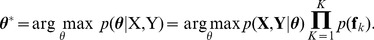
(6)


This is equivalent to seeking the minimal energy
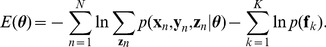
(7)


The set of transformations 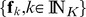 will be directly obtained from the optimal solution 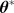, and the latent variables 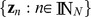 determine the inliers. In the next section, we show how to solve the estimation problem using an EM approach.

### The EM Algorithm

There are several ways to estimate the parameters of the mixture model, such as EM algorithm, gradient descent, and variational inference. The EM algorithm [29] is a general technique dealing with the existence of latent variables. It alternates with two steps: an expectation step (E-step) and a maximization step (M-step). We follow standard notations [34] and omit some terms that are independent of 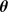. Denote 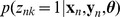 by 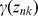, considering the negative log posterior function, i.e. equation (7), the complete-data log posterior is then given by
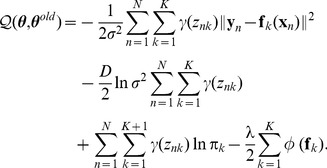
(8)


*E-step*: We use the current parameter values 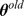 to find the posterior distribution of the latent variables, which can be found by applying Bayes' rules
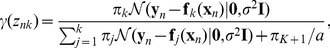
(9)where 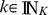 and 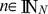. The posterior probability 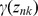 indicates to what degree the 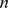-th sample agrees with the current estimated transformation 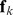.

*M-step*: We determine the revised parameter estimate 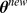 as follows: 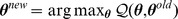. Taking derivative of 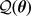 with respect to 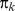 and 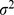, and setting them to zero, together with equation (2), we obtain
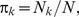
(10)

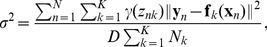
(11)where 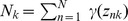.

Next we consider the terms of 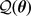 that are related to 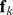. We obtain a regularized risk functional as [8]:
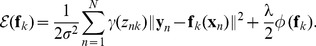
(12)


We model 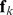 by requiring it to lie within a specific functional space 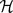, namely a vector-valued reproducing kernel Hilbert space (RKHS) [35]. We define the RKHS 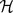 by a diagonal Gaussian kernel [8]: 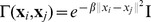. For the smoothness functional 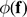, we use the square norm, i.e., 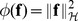. Therefore, we have the following representer theorem [36].

#### Theorem 1

*The optimal solution of the regularized risk functional (12) is given by*

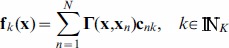
(13)
*with the coefficient set* 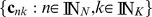 *determined by a linear system*

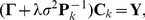
(14)
*where* 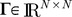 *is the kernel matrix with the 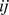-th entry* 
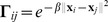
, 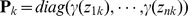 *is a diagonal matrix, and* 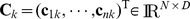.

*Proof*. The kernel 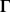 has the following reproducing property, for all 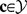 and 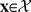


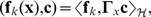
(15)where 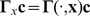. For any given reproducing kernel 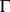, a unique RKHS can be defined as
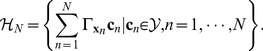
(16)


For further details about the reproducing property we refer the readers to [36]–[38].

Let 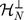 be a subspace of 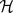,
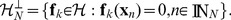
(17)


Form the reproducing property, i.e. equation (15), 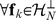


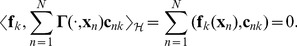
(18)


Thus 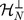 is the orthogonal complement of 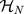; then every 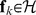 can be uniquely decomposed in components along and perpendicular to 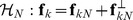, where 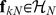 and 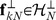. Since by orthogonality 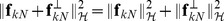 and by the reproducing property 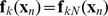, the regularized risk functional then satisfies
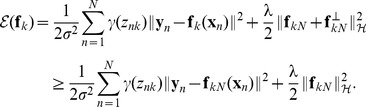
(19)


Therefore, the optimal solution of the regularized risk functional (12) comes from the space 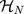, and hence has the form (13). To solve for the coefficients, we rewrite the regularized risk functional in the following matrix form:
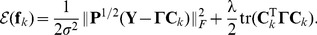
(20)where 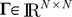
is the kernel matrix with the 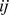-th entry 
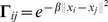
, 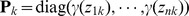 is a diagonal matrix, 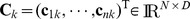 is the coefficient matrix, 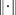 is the Frobenius norm, and 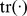 denotes the trace. Taking the derivative of the last equation with respect to 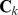 and setting it to zero, we obtain the linear system in equation (14). Thus the coefficient set 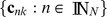 of the optimal solution 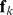 is determined by the linear system (14).

Once the EM algorithm converges, we then obtain the set of transformations 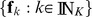. Besides, with a predefined threshold 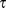 we obtain the inliers 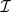 as well:
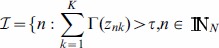
(21)


We summarize our method in Table 1. Since our robust point matching method is based on a mixture model, we named our method *RPM-MM*.

**Table 1:** Algorithm 1: The RPM-MM Algorithm.

**Input**: Correspondences 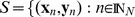 , parameters 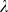 , 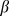 , 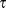
**Output**: Transformations 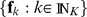 , inliers 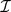
1. Initialize 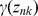 , 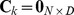 ;
2. Compute 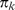 , 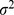 by equations (10) and (11);
3. Set the constant 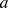 and compute the kernel matrix 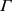 ;
4. **repeat**
5. *E-step*:
6. Update 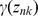 by equation (9);
7. *M-step*:
8. Update 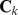 by solving linear system (14);
9. Compute 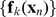 by equation (13);
10. Update 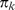 and 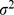 by equations (10) and (11);
11. **until** 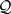 converges;
12. 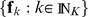 and 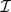 are determined by equations (13) and (21).

### Fast Implementation

Solving the transformation 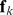 merely requires to solve the linear system (14). However, for large values of 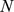, it may pose a serious problem due to heavy computational (e.g. scales as 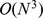) or memory (e.g. scales as 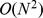) requirements, and, even when it is implementable, one may prefer a suboptimal but simpler method. To address this problem, in this section we provide a fast implementation based on a similar kind of idea as the subset of regressors method [17], [39].

Rather than searching for the optimal solution in 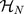 i.e., equation (16), we use a sparse approximation and search a suboptimal solution in a space 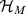 with much less basis functions defined as
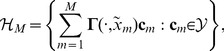
(22)and then minimize the regularized risk functional over all the sample data. Here 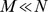 and we choose the point set 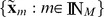 as a random subset of 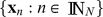 according to [17]. There, it was found that simply selecting an arbitrary subset of the training inputs performs no worse than more sophisticated methods. According to the sparse approximation, we search a solution with the form
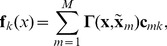
(23)with the coefficients 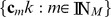 determined by a linear system
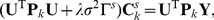
(24)where 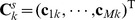 is the coefficient matrix, 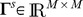 with the 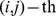 element 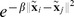, 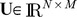 with the 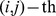 element 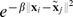.

In contrast to the optimal solution given by the representer theorem, which is a linear combination of the basis functions 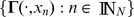, the suboptimal solution is formed by a linear combination of arbitrary 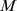-tuples of the basis functions. Generally, this sparse approximation will yield a vast increase in speed and decrease in memory requirements with negligible decrease in accuracy. Compared with the original algorithm shown in Algorithm 1, the fast version solves a different linear system (24) in Line 8.

### Computational Complexity

For the RPM-MM algorithm, the corresponding kernel matrix 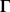 is of size 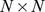. According to Theorem 1, we need to solve a linear system (14) for each transformation 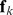. The time complexity is 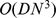, which is the most time-consuming step in the algorithm. As a result, the total time complexity of our algorithm is 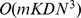, where 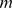 is the number of EM iterations. In our current implementation, we just use the Matlab “
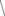
” operator, which implicitly uses Cholesky decomposition to invert a matrix. The space complexity of RPM-MM scales like 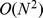 due to the memory requirements for storing the kernel matrix 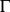.

For the fast implementation, the corresponding kernel matrix is of size 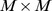, where 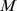 is the number of basis functions used for sparse representation. Then the time complexity is reduced to 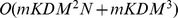, and the space complexity is reduced to 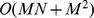. Typically, in point matching problems, the number of the point matches 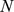 is in the order of 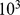, and the required number of basis function 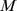 is in the order of 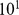. Therefore, both the time and space complexities can be simply written as 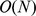. This is significant for large datasets. Our experiments demonstrate that the fast version is much faster than the original RPM-MM algorithm with negligible performance degradation.

### Extension to Non-Rigid Point Set Registration

Point set registration aims to align two point sets 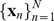 (the model point set) and 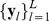 (the target point set). Typically, in the non-rigid case, it requires estimating a non-rigid transformation 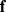 which warps the model point set to the target point set. Moreover, for point sets with multi-layer motion, it may need multiple transformations to achieve satisfying results. Recall that our RPM-MM method is able to generate a set of non-rigid transformations with adherence to a set of point correspondences. Therefore, it could be used to recover the transformation(s) between two point sets with a set of putative correspondences. Next we discuss how to establish initial correspondences.

Typically, for a pure point matching problem, the appearance information is not available. In general, if the two point sets have similar shapes, the corresponding points have similar neighborhood structures which could be incorporated into a feature descriptor. Thus finding correspondences between two point sets is equivalent to finding for each point in one point set (e.g., the model) the point in the other point set (e.g., the target) that has the most similar feature descriptor. Fortunately, the initial correspondences need not be very accurate, since our method is robust to noise and outliers. Inspired by these facts, we use shape context [40] as the feature descriptor, using the Hungarian method for matching with the 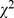 test statistic as the cost measure.

After we get the rough correspondences between two point sets based on their shape features, we fit a set of transformations and use them to warp the model points. To this end, we need to determine the attribution of each model point (i.e., which component of the mixture model a model point belongs to). First, according to the matching results based on the rough correspondences, we can determine the attributions of the inliers; for the rest of the model points, we attribute them to their nearest neighbors which already have attributions. The two steps of estimating correspondences and transformations are iterated to obtain a reliable result. In this paper, we use a fixed number of iterations, typically 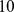 but more when there are large degradations on the data. We summarize our non-rigid point set registration method in Table 2.

**Table 2:** Algorithm 2: Non-Rigid Point Set Registration via RPM-MM.

**Input**: Two point sets 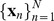 , 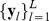
**Output**: Aligned model point set 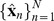
1. Compute feature descriptors for the target point set 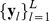 ;
2. **repeat**
3. Compute feature descriptors for the model point set 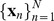 ;
4. Estimate the initial correspondences based on feature descriptors;
5. Solve the transformations 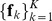 by using RPM-MM;
6. Warp the model point set according to the transformations 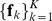 ;
7. Update the model point set by using the warped model point set;
8. **until** reach the maximum iteration number;
9. 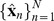 is given by the warped model point set in the last iteration.

### Implementation Details

The performance of point matching algorithms typically depends on the coordinate system in which points are expressed; here we use data normalization to control for this. More specifically, we perform a linear re-scaling of the correspondences so that the points in the two sets both have zero mean and unit variance. Furthermore, we define the transformation 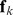 as the initial position plus a displacement function 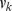: 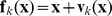 [11], and solve for 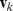 instead of 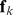. This can be achieved simply by setting the output 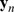 to be 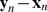. The use of displacement function achieves more robustness.

The EM algorithm is well known to converge to a local maximum. To initialize the EM iteration, we first use the K-means algorithm to cluster the correspondences into 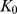 clusters. To this end, we convert the correspondences into a set of motion field samples 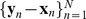 and do clustering on them. The first 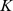 largest clusters are considered as inliers of the 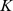-component mixture model in our approach, and then are used to initialize 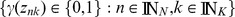. In our evaluation, we set 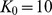, and 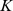 can be set adaptively according to the cardinalities of the clusters. For instance, if the ratio of the cardinalities between a certain cluster and the largest cluster is bigger than a threshold, e.g., 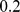, then it will be preserved as initial inliers, typically 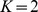 or 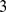.

There are mainly three parameters in our algorithm: 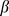, 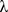 and 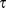. Parameters 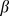 and 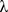 both reflect the amount of smoothness regularization. Parameter 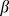 determines how wide the range of interaction between correspondences. Parameter 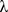 controls the trade-off between the closeness to the data and the smoothness of the solution. Parameter 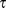 is a threshold, which is used for deciding the correctness of a correspondence. In general, we find our method to be very robust to parameter changes. We set 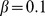, 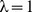, and 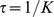 throughout the experiments. The constant 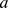 in the uniform distribution is set to the volume of the output space (e.g., the bounding box of the points) after data normalization, typically 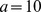. Besides, for the fast implementation, the number 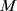 of basis functions used for sparse approximation is fixed to 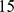.

## Experimental Results

In order to evaluate the performance of our algorithm, we conducted two types of experiments: i) sparse image feature correspondence on 2D images and 3D surfaces; ii) non-rigid shape matching on synthetical 2D datasets.

### Results on Sparse Feature Correspondence

**Figure 2:**
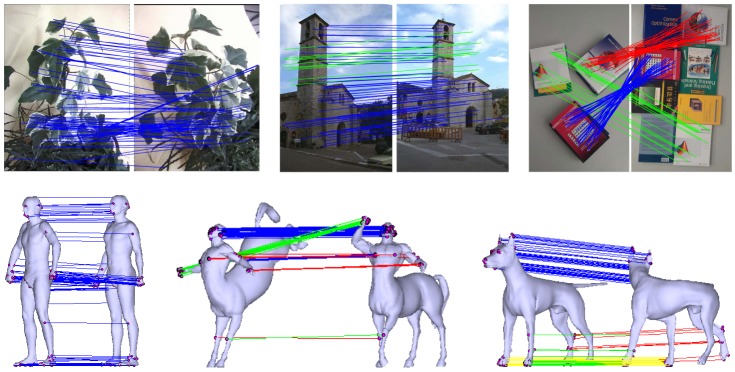
Matching results on 2D image pairs (*Tree*, *Church* and *Books*) and 3D surface pairs (*Person*, *Centaur* and *Dog* [42]). Different color denotes different component of the mixture model. For visibility, in the image, we only show at most 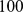 randomly selected elements of the preserved correspondences by our method.

We present a few representative matching results on both 2D image pairs and 3D surface pairs, as shown in Fig. 2. For the 2D images, the first two pairs (i.e., *Tree* and *Church*) are wide baseline images which are public available [41]. The third pair (i.e., *Books*) contains some shared content which is taken by ourselves. It often occurs in image or object retrieval. The 3D surface pairs (i.e., *Person*, *Centaur* and *Dog*) are objects with different amounts of non-rigid deformation, which come from a surface correspondence benchmark [42]. In our evaluation, we use the SIFT [2] and MeshDOG/MeshHOG [43] to establish putative correspondences for 2D and 3D cases, respectively.

The match correctness is determined as follows. For the 2D images, a method combining subjectivity and objectivity is considered. We first fit the epipolar geometry (e.g., fundamental matrix) by RANSAC and use it to determine the match correctness. We further confirm them artificially. Although the judgment of correct match and mismatch seems arbitrary, we make the benchmark before performing experiments to ensure objectivity. For the 3D surfaces, the ground truth correspondences are supplied by the dataset.

The experimental results are evaluated by precision and recall, where the precision is defined as the ratio of the preserved inlier number and the preserved correspondence number, and the recall is defined as the ratio of the preserved inlier number and the inlier number contained in the putative correspondences. We compare our RPM-MM algorithm with other four methods which remove outliers from given putative point correspondences, such as RANSAC [3], ICF [5], GS [13], and VFC [8]. We implement ICF and tune all parameters accordingly to find optimal settings. For RANSAC, GS and VFC, we implement them based on the publicly available codes. Throughout all the experiments, five algorithms' parameters are all fixed.

The results of our RPM-MM are presented in Fig. 2, we see that for an image pair with relatively simple structures, such as *Tree* or *Person* which involves a small amount of rotation, viewpoint change, or non-rigid deformation, the mixture model in our algorithm then degenerates and contains only one component. In this case Our RPM-MM algorithm is equal to a normal non-parametric model-based method, e.g., VFC [8]. For images with large view point change or non-rigid deformation, the mixture model will contain multiple components to capture multi-layer motion, such as *Church*, *Books*, *Centaur* and *Dog*. Note that in the image pair of *Church*, our RPM-MM preserves the correspondences on the sky; this is very useful since removing inliers outstanding in depth tend to make the recovery of epipolar geometry unstable and ill conditioned [44]. Moreover, the matching result of *Books* shows our method's capability in image retrieval.

We further quantitatively compare our RPM-MM to four state-of-the-art point matching algorithms: RANSAC, ICF, GS and VFC. Tables 3 and 4 report the 2D and 3D results respectively. As shown in Table 3, ICF and VFC have low recalls when the scene contains large discontinuities. In fact, they tend to preserve typically one major component (i.e., the matches marked by blue lines in Fig. 2) or two components of the correspondences. RANSAC has satisfactory performance when the relationship of correspondence is rigid, e.g., epipolar geometry. But it can not work in the non-rigid case, e.g., *Books*. The graph matching based method GS generally can obtain better performance than ICF and VFC in case of large discontinuity, e.g., *Books*. But its recalls are still relatively low compared to our RPM-MM. In Table 4 we only use VFC for comparison since the other three methods are not applicable for either the 3D case or non-rigid deformation. We again observe that VFC fails to keep most of the inliers under large non-rigid deformations. Our RPM-MM in general has the best precision-recall trade-off, and it is not affected by large non-rigid deformation or multi-layer motion. In addition, we also test the fast version of our method on these six image pairs, as shown in the last rows of Tables 3 and 4. The average number of putative correspondences on these six image pairs is about 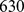, and the average run times of our RPM-MM and its fast implementation are about 6 s and 0.2 s on an Intel Core 2.0 GHz PC with Matlab code. We see that the use of sparse approximation leads to an essential speedup without much performance degradation.

**Table 3:** Performance comparison on 2D image pairs in Fig. 2: *Tree*, *Church*, and *Books*.

Inlier pct.	56.29 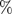	54.76 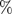	75.74 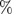
RANSAC [3]	(94.68, 94.68)	(94.52, 100.00)	-
ICF [5]	(92.75, 68.09)	(91.67, 63.77)	(91.24, 40.53)
GS [13]	(97.62, 87.23)	(91.78, 97.10)	(100.00, 82.48)
VFC [8]	(94.85, 97.87)	(98.33, 85.51)	(97.79, 70.44)
RPM-MM	(94.85, 97.87)	(97.14, 98.57)	(99.82, 98.05)
RPM-MM (fast)	(94.85, 97.87)	(95.77, 98.55)	(99.82, 98.23)

The pair denotes the precision-recall pair (%).

**Table 4:** Performance comparison on 3D surface pairs in Fig. 2: *Person*, *Centaur*, and *Dog*.

Inlier pct.	56.40 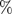	78.23 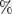	86.18 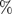
VFC [8]	(99.22, 98.46)	(99.53, 78.85)	(96.58, 82.65)
RPM-MM	(99.22, 98.46)	(97.11, 93.51)	(95.71, 96.27)
RPM-MM (fast)	(99.22, 98.46)	(97.04, 93.09)	(95.74, 96.40)

The pair denotes the precision-recall pair (%).

### Results on Shape Matching

**Figure 3:**
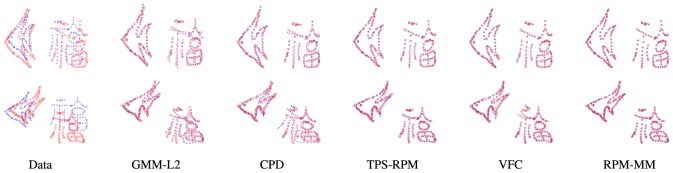
Point set registration results. The data contains two independent shape models with different movements and distortions. The goal is to align the model point set (blue pluses) onto the target point set (red circles). From left to right: model and target point sets, registration results of GMM-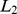 [22], CPD [21], TPS-RPM [20], VFC [8] and RPM-MM.

We next evaluate our RPM-MM on the non-rigid point set matching problem, more specifically, shape matching. For the dataset, we choose the same synthesized data as in [20]. The data consists of two different shape models, where the first model consists of 96 points representing a fish shape, and the second model is a more complex pattern consisting of 108 points representing a Chinese character. We combine the two shapes to generate a model point set, i.e., the blue pluses as shown in Fig. 3. To get a target point set, we apply two randomly generated non-rigid transformations on the model point set and warp the two shape models respectively, i.e., the red circles as shown in Fig. 3. The model is warped through progressively larger degrees of non-rigid warpings, and we generate 100 samples in total.

Fig. 3 presents some registration results of our RPM-MM and four other state-of-the-art registration algorithms: GMM-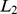 [22], CPD [21], TPS-RPM [20] and VFC [8], which are implemented using publicly available codes. In the first row, the data contains a relatively slight deformation; in this case, it is possible to search a single transformation which approximates the two-layer motion well. Therefore, all the five algorithms are able to generate satisfying results. However, the matching performance degrades gradually as the degree of deformation increases. In the second row, the data contains a relatively large deformation; in this case, just one transformation cannot capture the two-layer motion, and hence the matching performance degrades. By contrast, our RPM-MM uses a mixture model which interpolates a set of transformations, and hence is robust to the two-layer motion. Therefore, it still can produce an almost perfect alignment, as shown in the last column of Fig. 3.

**Figure 4:**
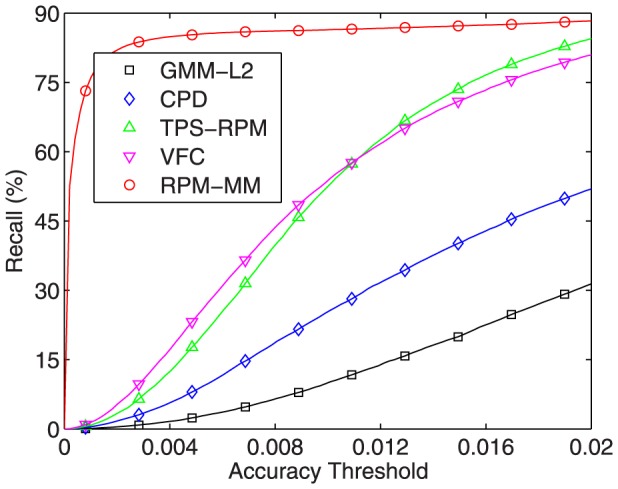
Performances of non-rigid point set registration algorithms over 100 samples.

To provide a quantitative comparison, we report the registration results of the five algorithms on all the 100 samples. We compute the recall as the metric used in [22]. Here the recall, or true positive rate, is defined as the proportion of true positive correspondences to the ground truth correspondences and a true positive correspondence is counted when the pair falls within a given accuracy threshold in terms of pairwise distance, e.g., the Euclidean distance between a point in the warped model and the corresponding point in the target. Fig. 4 plots the recall curves of the five algorithms on all the 100 samples. We see that VFC and TPS-RPM perform much better than CPD and GMM-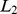, and our RPM-MM is far better than the other four methods. This is not surprise since our RPM-MM interpolates multiple transformations to capture multi-layer motion while the other four algorithms interpolate just a global transformation. That is to say, for point sets with multi-layer motion, it is necessary to interpolate multiple local transformations (as in our RPM-MM) rather than only one global transformation to achieve accurate results. We also tested the fast version of our algorithm on this dataset and obtained almost the same recall curve as the original version, here we omit it in the figure for clarity.

## Conclusion

Within this paper, we have proposed and studied a new robust point matching algorithm based on a mixture model (RPM-MM). It interpolates a set of transformations to fit different layers of the motion correspondence by an iterative EM algorithm, and hence establish reliable correspondence between two images. Quantitative comparisons on both sparse feature correspondence and shape matching demonstrate that our algorithm outperforms state-of-the-art point matching methods, especially when the motion of the scene contains large discontinuities.
